# Subduction of a low-salinity water mass around the Xisha Islands in the South China Sea

**DOI:** 10.1038/s41598-018-21364-3

**Published:** 2018-02-15

**Authors:** Zhida Huang, Wei Zhuang, Hailong Liu, Jianyu Hu

**Affiliations:** 1 State Key Laboratory of Marine Environmental Science, College of Ocean and Earth Sciences, Xiamen University, Xiamen, 361102 China; 2 Key Laboratory of Coastal and Wetland Ecosystems of Ministry of Education, College of the Environment and Ecology, Xiamen University, Xiamen, 361102 China; 3 State Key Laboratory of Numerical Modeling for Atmospheric Sciences and Geophysical Fluid Dynamics, Institute of Atmospheric Physics, Chinese Academy of Sciences, Beijing, 100029 China; 4 College of Earth Sciences, University of Chinese Academy of Sciences, Beijing, 100049 China

## Abstract

Based on three climatologically observed temperature and salinity datasets (i.e., GDEM-V3, SCSPOD14 and WOA13), this paper reports a low-salinity (~34.32) water mass in the subsurface-to-intermediate layer around the Xisha Islands in the South China Sea. This water mass mainly subducts from the surface layer into the intermediate layer, characterized by a relatively low potential vorticity tongue extending from the bottom of mixed layer to the thermocline, and accompanied by a thermocline ventilation in spring (especially in April). The potential dynamics are the joint effects of negative wind stress curl, and an anticyclonic eddy triggered by the inherent topographic effect of the Xisha Islands, reflecting that downward vertical motion dominates the subduction. Despite lacking of the homogenous temperature and density, the low-salinity water mass is to some extent similar to the classic mode water and can be regarded as a deformed mode water in the South China Sea.

## Introduction

**Figure 1:**
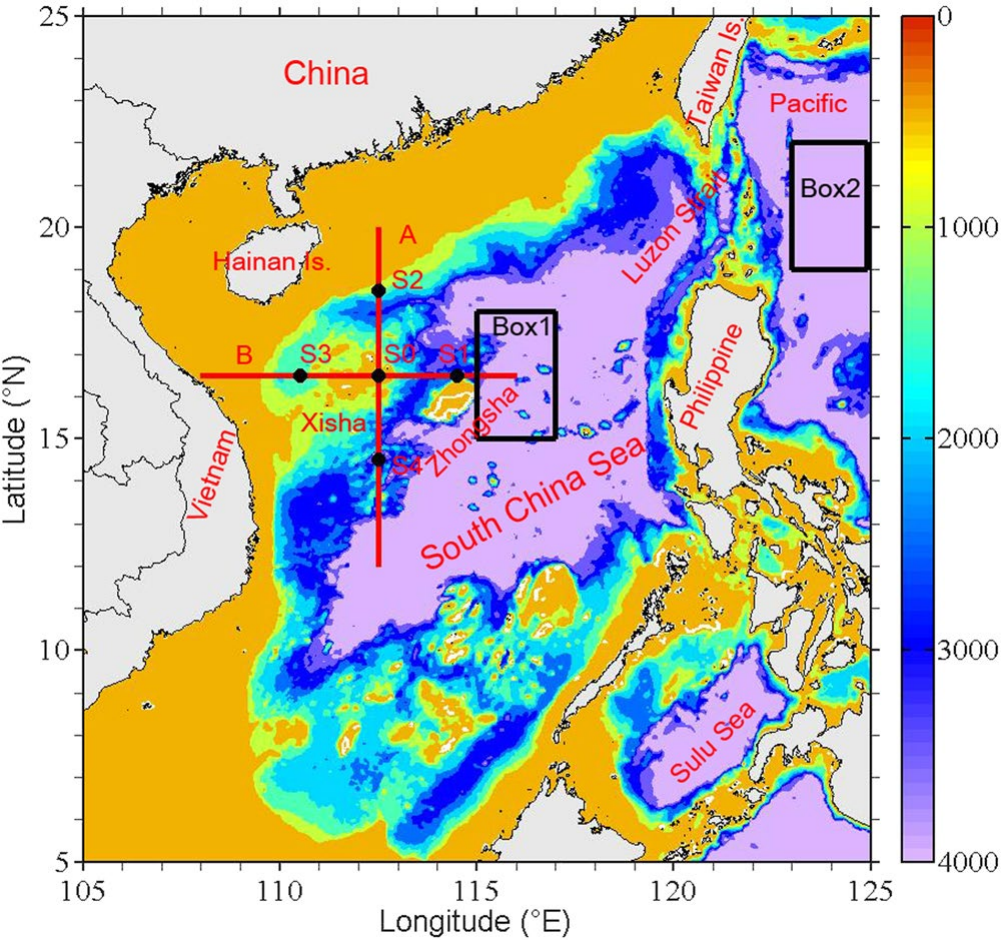
Topography around the South China Sea from the ETOPO2 bathymetry (in m). The two red lines (12°N–20°N, 112.5°E; 16.5°N, 108°E–116°E) indicate Transects A and B across the Xisha Islands. Five black dots S0–S4 are used to study the T-S characteristics. Box 1 (15°N–18°N, 115°E–117°E) and Box 2 (19°N–22°N, 123°E–125°E) are selected to plot the mean T-S diagrams for the SCS water and NP water, respectively. The figure was made using MATLAB R2012a (http://www.mathworks.com/).

The South China Sea (SCS) is the largest semi-enclosed marginal deep sea located west of the North Pacific (NP) (Fig. 1), and its circulation patterns are associated with East Asian monsoon and Kuroshio intrusion. Much work has been done on a variety of dynamic processes in the SCS, including the vertical three-layer (cyclonic-anticyclonic-cyclonic) circulation structure, the SCS meridional overturning circulation, water exchanges in the Luzon Strait, mesoscale eddies and internal waves^1–13^. Analysis of Levitus dataset indicated a thermocline ventilation in the northern SCS in winter, leading to the detrainment of surface mixed layer water into the seasonal thermocline and subsequent southward migration of this water mass due to basin-scale cyclonic circulation^14^. However, whether the mixed layer water further subducts into the main thermocline remains unclear.

The large-scale subduction is the transfer of fluid from the surface mixed layer of the ocean into the interior thermocline, which is quantified by the subduction rate measured by the volume flux of fluid per unit area entering the thermocline from the mixed layer^15,16^. The subduction process is achieved through either shallowing of the mixed layer, lateral transfer of fluid across the sloping mixed layer base, or through vertical motion. In an eddying ocean, the net subduction rate incorporates both Eulerian-mean and eddy contributions^16–18^. The additional eddy-induced subduction can be regarded as the rectified transfer of a water mass from the mixed layer into the thermocline by an eddy-induced “bolus” velocity^19^. Recently, observations presented that subduction caused by an anticyclonic eddy (AE) is comparable in magnitude to that by the mean flow^18^. These suggest that eddies play a significant role in the total subduction. Similar to the NP, lots of mesoscale AEs in the SCS^9,10^ will favor for the subduction formation.

In our study region (Fig. 1), our goals are to explore a subduction of low-salinity water mass which has been rarely reported before, and to preliminarily analyze the potential dynamic mechanisms for its formation, by using climatologically observed datasets. The present study will provide clearer insight into the circulation dynamics, ecological effect and climate change in the SCS.

## Results

### Low-salinity water mass around Xisha Islands

The climatologically observed dataset is collected from the U. S. Navy Generalized Digital Environment Model version 3 (GDEM-V3), with a horizontal resolution of 0.25° × 0.25°, and a vertical grid of 78 standard layers ranging from the surface to 6600 m^20^. Detailed information about the GDEM-V3 is provided in Methods section.

**Figure 2:**
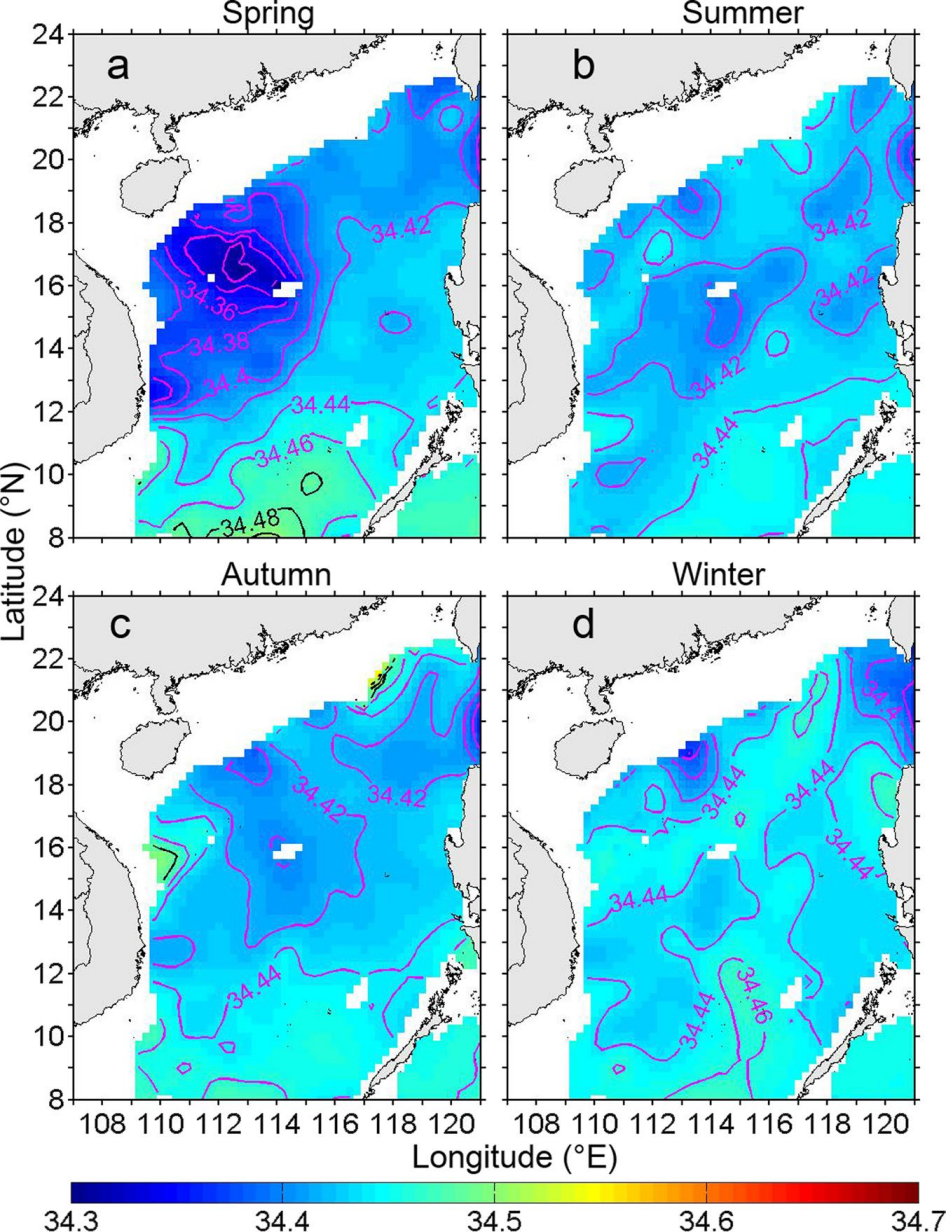
Seasonal distribution of salinity in the intermediate layer (500 m) for: (**a**) spring, (**b**) summer, (**c**) autumn and (**d**) winter, based on the GDEM-V3 dataset^20^. Spring is defined as March, April and May average, and so on. The contour interval is 0.02. The purple contours highlight the isohaline with value equal to or less than 34.46. The figure was made using MATLAB R2012a (http://www.mathworks.com/).

Figure 2 shows the salinity in the intermediate layer (i.e., 500 m). In spring, there are two relatively low-salinity areas in the western SCS (Fig. 2a), one is located near the Xisha Islands (XS) with minimum salinity of 34.32, and the other is located east of Vietnam with that of 34.36. Near the XS, the 34.34 isohaline indicates that the relatively low-salinity area has a size of at least 200 km. In spring, the subsurface layer (i.e., 150 m; Fig. S1) also has two relatively low-salinity areas, which correspond to those in the intermediate layer (Fig. 2a). In Fig. S1, the minimum salinity values are about 34.44 and 34.46 near the XS and east of the Vietnam, respectively. Different from spring, the relatively low-salinity structures are not presented in other three seasons (Fig. 2 and S1).

The minimum salinity values in the subsurface (i.e., 150 m) and intermediate layers near the XS are 0.02 and 0.04 lower than those east of Vietnam (Fig. 2a and S1a), respectively. More importantly, the minimum salinity in the intermediate layer in the northeastern SCS is mainly between 34.40 and 34.42, which is about 0.1 higher than that around XS (Fig. 2a). This suggests that the low-salinity NP Intermediate Water intrusion is confined in the northeastern SCS and cannot account for the appearance of the low-salinity structure in the intermediate layer around the XS.

**Figure 3:**
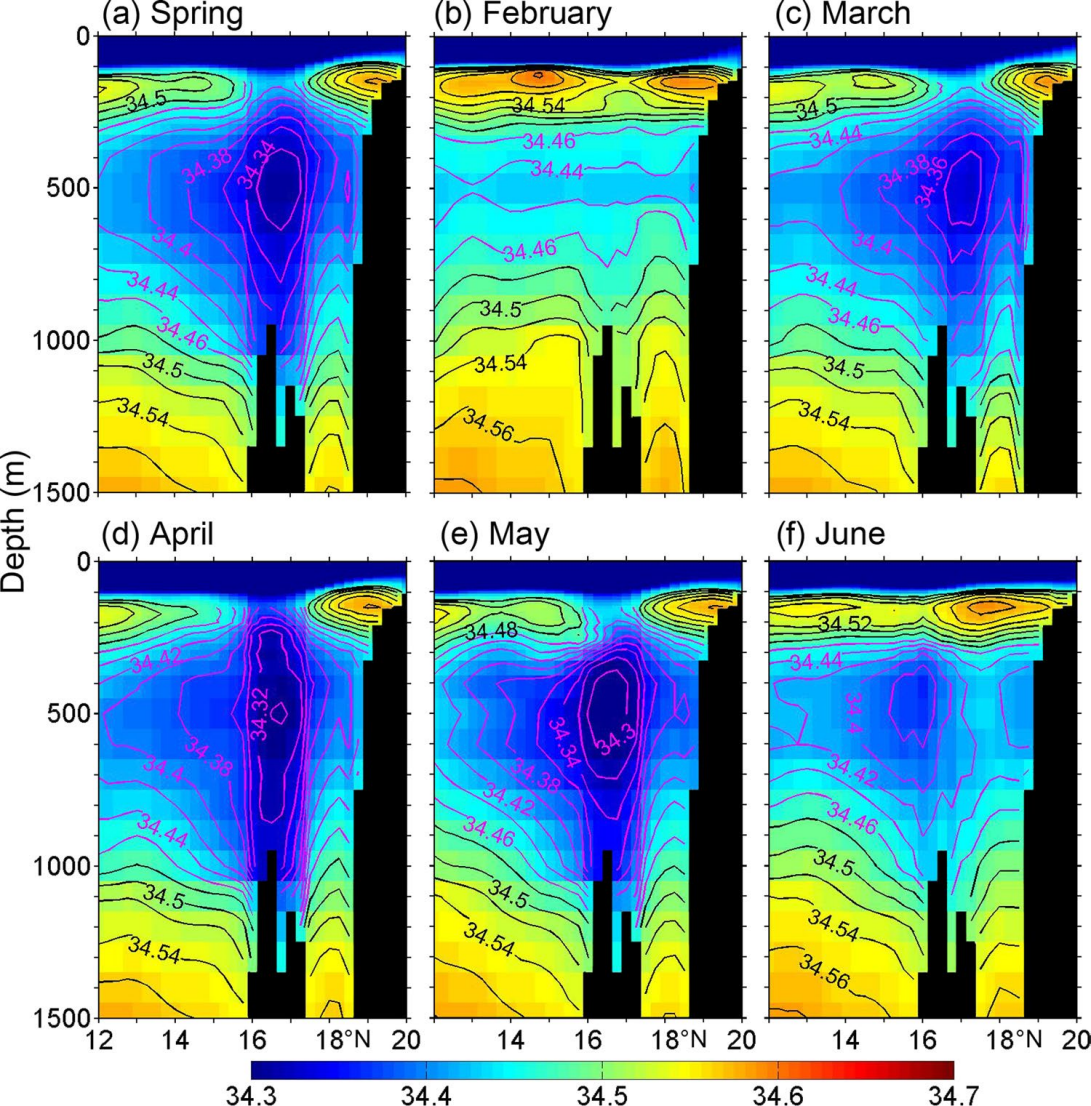
Sectional distribution of salinity for Transect A in: (**a**) Spring, (**b**) February, (**c**) March, (**d**) April, (**e**) May and (**f**) June, with the contour interval of 0.02. Spring is defined as March, April and May average. The purple contours highlight the isohaline with value equal to or less than 34.46. The black shades indicate the topography extracted from the GDEM-V3 dataset^20^. The figure was made using MATLAB R2012a (http://www.mathworks.com/).

In order to further present the vertical structure of the low-salinity water around the XS, Fig. 3a shows the vertical salinity distribution between 12°N and 20°N along 112.5°E in spring (Transect A in Fig. 1). In spring, the low-salinity water beneath the subsurface salinity maximum layer ranges from 400 to 700 m around the XS, with the minimum salinity smaller than 34.32 (Figs 1 and 3a). The salinity evolution shows that the low-salinity core is located at about 500 m depth between 16°N and 17°N, with salinity of about 34.34, 34.30 and 34.30 in March, April and May, respectively (Fig. 3c–e). Especially in April, it is clear that the 34.32 isohaline is distributed between 250 and 800 m and the 34.34 isohaline between 200 and 1000 m, which indicates that there exists significant vertical homogeneity of low-salinity water. Near the XS, the maximum salinity value is about 34.38 in the subsurface layer in April which is smaller than that in other months. Compared to Transect A, no remarkable difference is found along the Transect B except for the low-salinity structure slight tilting near the XS (Fig. S2). Clearly, a negative anomaly signal propagates downward to the intermediate layer near the XS in spring, especially in April with the minimum anomaly value exceeding −0.10 between 50 and 1000 m (Fig. S3). South of the XS, the largest negative signal occurs between 50 and 100 m, and weakens from March to May (Fig. S3a–c).

The T-S diagrams at stations S1–S4 (Fig. 1 and S4) in April are close to the SCS mean, except that the former has lower salinity than the latter in the intermediate layer. It is clear that station S0 presents homogeneous low-salinity with a value of 34.30 between 200 and 900 m, which is different from the other stations. At the depth of about 100 m or so, the salinity is about 34.20 at station S0, while those are about 34.40, 34.50, 34.40 and 34.30 at stations S1–S4, respectively. Furthermore, the surface salinity at station S0 is 0.10 greater than that at station S4, but at least 0.10 lower than that at stations S1–S3. At station S0, the salinity in the subsurface layer (e.g., 150 m) is about 0.10 greater than that between 200 and 900 m.

As shown above, the GDEM-V3 dataset demonstrates that a relatively low-salinity water mass appears in the intermediate layer near the XS in spring, especially for April (Figs 2 and 3). We also check two other climatological datasets, the SCS Physical Oceanographic dataset (SCSPOD14^21^) and the World Ocean Atlas 2013 (WOA13^22,23^). Detailed information about the SCSPOD14 and WOA13 are given in the Methods section. In spring, results of these two datasets (especially the SCSPOD14) also reflect the low-salinity property around the XS (Fig. S5), which are in agreement with the GDEM-V3. The above consistency among these three climatological datasets further verifies the existence of low-salinity structure in the intermediate layer near the XS. Therefore, we further investigate the dynamics for its formation.

### Formation mechanism

Potential formation mechanism for the low-salinity water mass is now investigated by geostrophic current analyses. The altimeter-based surface geostrophic currents are provided by the Archiving, Validation, and Interpretation of Satellite Oceanographic data (AVISO; see Methods for more information). We also calculate the geostrophic currents based on the GDEM-V3 dataset (see Methods for more detailed information).

**Figure 4:**
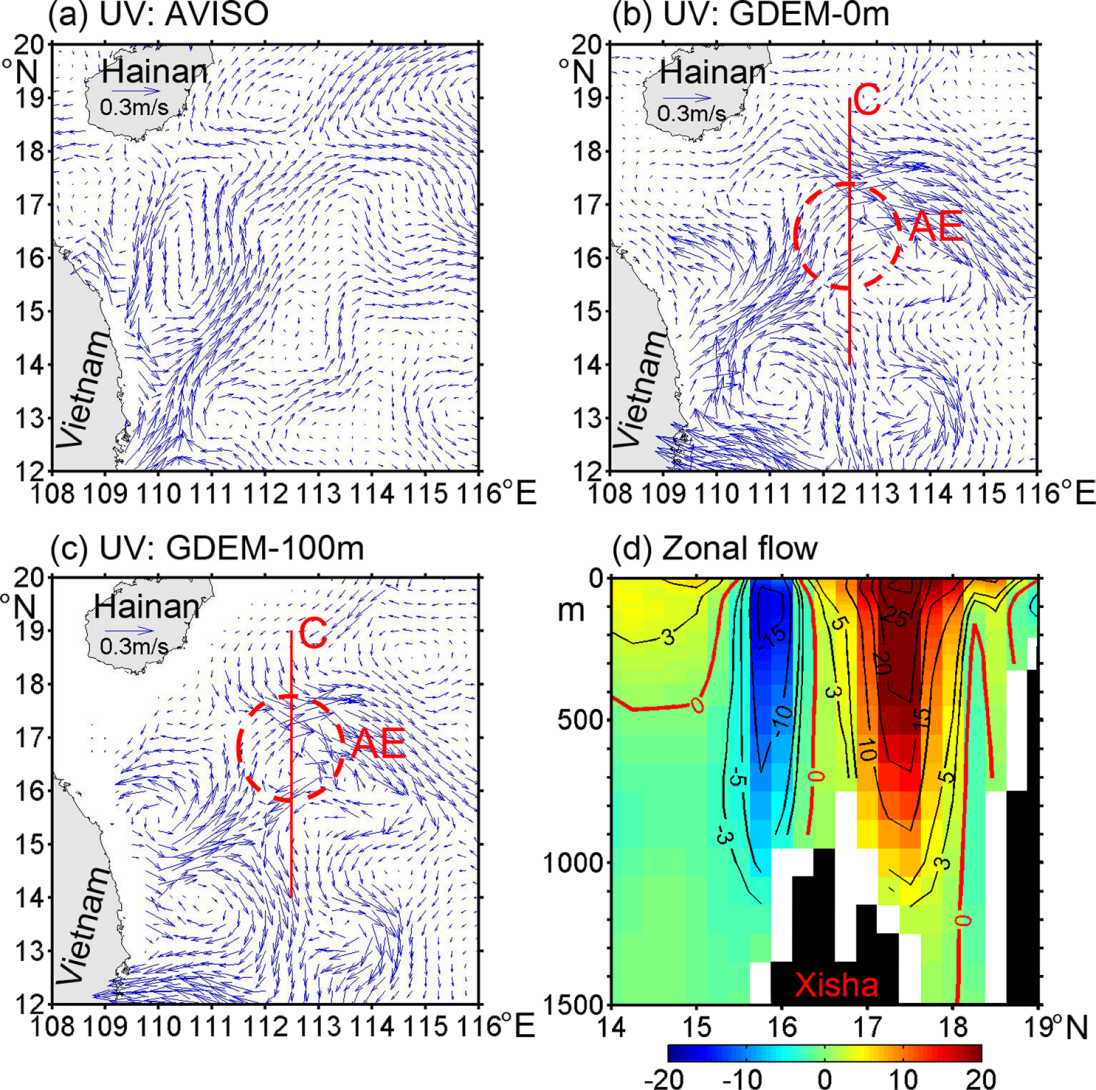
Surface geostrophic current patterns in April based on (**a**) AVISO from 1993 to 2016 and (**b**) GDEM-V3 products^20^. (**c**) Geostrophic current pattern in the 100 m layer. (**d**) Zonal flow (cm s^−1^) along Transect C (shown as a red line in (**b**) or (**c**), which is similar as Transect A except for 14°N–19°N). The red dashed circle indicates an AE. The geostrophic currents in (**b–c**) are calculated with a reference to 2000 m. Positive (negative) values denote eastward (westward) flow. The red curves highlight the contour value of 0. The black shades indicate the topography extracted from the GDEM-V3 dataset^20^. The figure was plotted using MATLAB R2012a (http://www.mathworks.com/).

In the surface layer, both AVISO and GDEM-V3 datasets in April present that the western boundary current flows away from east of Vietnam, moves northeastward to the XS, and then turns eastward (Fig. 4a,b), indicating that there is no much difference between the two datasets in terms of large-scale circulation pattern. Different from the AVISO, the GDEM-V3 indicates that a mesoscale AE (red dashed circle in Fig. 4b,c) with a size of about 200 km exists near the XS. Such an AE seems to exist almost every spring^24,25^.

Zonal flow structure along Transect C (14°N–19°N, 112.5°E; red line in Fig. 4b,c) further clarifies the vertical structure of AE which exhibits westward flow of 15 cm s^−1^ between 50 and 250 m south of the XS and eastward flow of 20 cm s^−1^ between 0 and 450 m north of the XS (Fig. 4d). On the whole, the zonal flow structure is symmetric from the 0 contour line appearing at 16.5°N just above the XS topography, which is consistent with the AE (Fig. 4b–d). The zonal flows have values of −10 and −5 cm s^−1^ at 500 and 1000 m south of the XS, respectively. This reflects that the vertical extent of the AE is at least 1000 m.

In the following, we examine the potential vorticity (PV) properties and ventilation phenomenon for further investigating the formation mechanism. Following the work of Lin *et al*.^26^, the PV is defined as:

1 $$PV=-\frac{f}{\rho }\cdot \frac{\partial \rho }{\partial z}-\frac{\zeta }{\rho }\cdot \frac{\partial \rho }{\partial z}$$

where *f* is the Coriolis parameter, $\zeta =\frac{\partial v}{\partial x}-\frac{\partial u}{\partial y}$ is the relative vorticity, *ρ* is the potential density. The PV is composed of the planetary potential vorticity (PPV) and relative potential vorticity (RPV). Along Transect C, in April the PV values are less than the PPV near the XS (Fig. S6), which is resulted from RPV corresponding to the AE (Fig. 4b–d). Near the XS, below 200 m the concave isopycnal structure is determined by salinity with a value less than 34.35 (Fig. S7).

**Figure 5:**
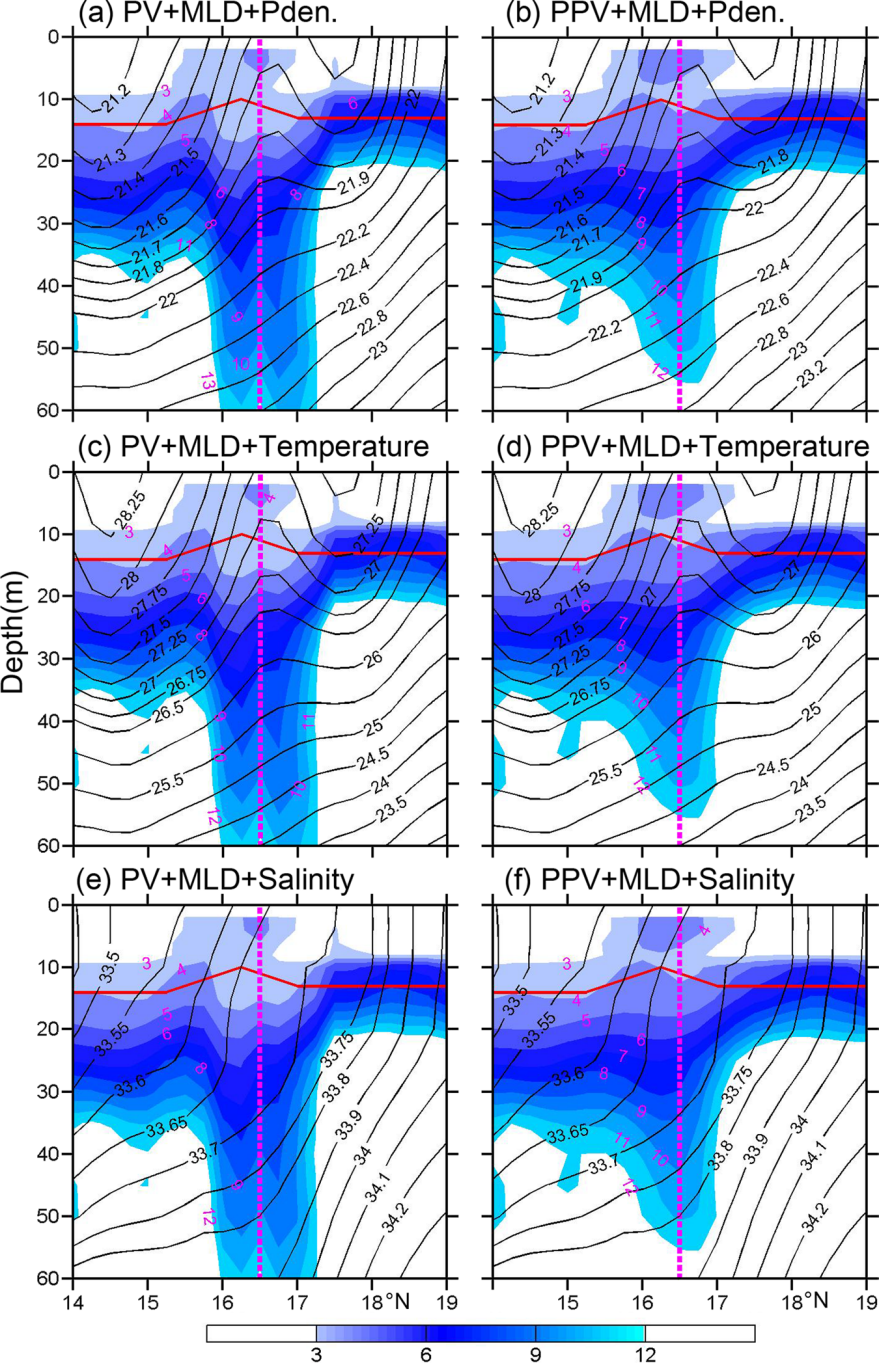
Distributions of potential vorticity (PV, left panels shaded in color; × 10^−10^ m^−1^ s^−1^) and planetary potential vorticity (PPV, right panels shaded in color; × 10^−10^ m^−1^ s^−1^) along Transect C in April. Potential density (kg m^−3^), potential temperature (°C) and salinity are shown in the top, middle and bottom rows, respectively. The pink dashed line indicates the location of XS. The red line denotes the mixed layer depth (MLD). The figure was plotted using MATLAB R2012a (http://www.mathworks.com/).

Figure 5 shows the distributions of PV, PPV, mixed layer depth (MLD) and thermohaline structures along Transect C between 0 and 60 m in April. The MLD is defined as the depth at which the water density is 0.1 kg m^−3^ denser than the sea surface^27^. The MLD intersects with the outcrops of 21.3–22.2 isopycnals, 26.5 °C–28.0 °C isotherms and 33.5–34.0 isohalines, which reflects thermocline ventilation^14^. Although the MLD in our study region is about 15 m (Fig. 5) and one-tenth of that in the NP subtropical mode water formation region^17^, there exists a relatively low PV (PPV) tongue with a value of 4–12 × 10^−10^ m^−1^ s^−1^ vertically extending from the bottom of the MLD to the thermocline near the XS (Fig. 5). This kind of relatively low PV(PPV) tongue occurs largely along the outcrop lines, which is similar to that in the NP subtropical mode water formation region^17^.

Different from a general AE in the ocean, the core of AE has a doming of isopycnals with values between 21.7 and 22.0 kg m^−3^ in the upper layer (Figs 4b–d and 5a). From the topography distribution (Figs 1 and 3), the XS can be regarded as a seamount. The isopycnal doming over a seamount is generally called as Taylor cap^28^. Based on interactions among the steady flow, tides and seamount, Beckmann and Mohn^28^ pointed out two major dynamic mechanisms for the Taylor cap. One is the Taylor column formation caused by strong and steady impinging flow, the other is seamount-trapped waves of large amplitude which may generate an anticyclonic residual current through a process called non-linear rectification. Furthermore, Ikeda and Yamada^29^ presented that topographic Rossby waves exist over the Shatsky Rise (near 33°N, 159°E) due to interactions between seamount and mesoscale eddies. Near the XS, observations showed that the M_1_ and M_2_ tides have large amplitudes of about 26 and 16 cm, respectively^30^. Recently, long-term measurements of a moored Aanderaa current meter presented that topographic Rossby waves exist in our study region^31^. These suggest that seamount-trapped waves maybe an important factor for producing the AE. According to the PV conservation $(\frac{f+\varsigma }{H}$ = constant; *f* is the Coriolis parameter, $\varsigma$ the relative vorticity and *H* the water depth), it is easy to understand the dynamic process for the AE.

We further investigate the evolution of wind stress curl (WSC), by analyzing the Quick SCATterometer dataset during the period of 2000–2008 (Fig. S8). Our study region is dominated by the northeasterly, easterly and southeasterly wind vectors in March, April and May, respectively, which clearly indicate that spring is the transition period for monsoon (Fig. S8b–d). In March, there is a negative WSC core near the XS, with a value less than −5 × 10^−8^ N m^−3^ (Fig. S8b). Except for the coastal area, in April most parts of our study region are featured by negative WSCs (Fig. S8c). Compared with March, the negative WSC core with lower value (−10 × 10^−8^ N m^−3^) appears east of the XS in April. However, near the XS the WSC is positive (5 × 10^−8^ N m^−3^) in May (Fig. S8d), which is different from that in March and April. In order to estimate roles of the negative WSCs quantitatively, we calculate the Ekman pumping rate (${W}_{Ekman}$) by ${W}_{Ekman}=curl(\overrightarrow{\tau }/\rho f)$, where $\overrightarrow{\tau }$ is the wind stress, *f* is the Coriolis parameter, *ρ* is the sea water density. Near the XS, in April the WSCs are about between −5 and −10 × 10^−8^ N m^−3^ (Fig. S8c). If we take *f* = 4.2 × 10^−5^ s^−1^ and *ρ* = 1024 kg m^−3^, the ${W}_{Ekman}$ is about between 1.2 and 2.4 × 10^−6^ m s^−1^ and comparable to that in the main subduction points in the eastern subtropical NP mode water formation region (about 1.7 × 10^−6^ m s^−1^ from February to March)^32^. Therefore, it is clear that the negative WSCs produce relatively large downward Ekman pumping rate and provide another favorable condition for the subduction of low-salinity water mass in April. Considering that the negative WSCs occupy a much larger area over the northwestern SCS compared with the localized low-salinity water around the XS, the influence of wind-driven Ekman pumping is probably minor than AE.

## Discussion

**Figure 6:**
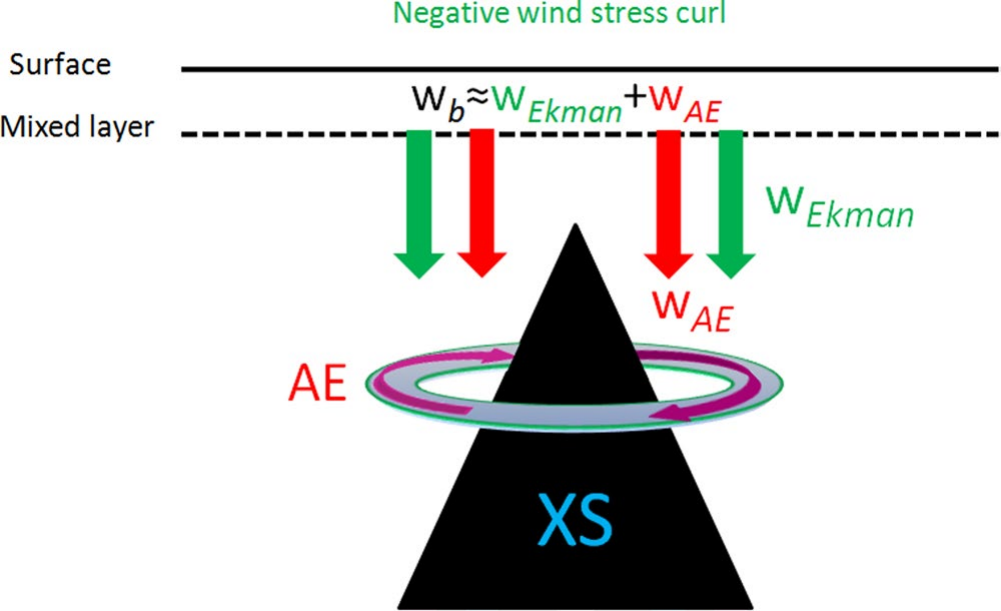
Schematic diagram showing the dynamic mechanisms for the subduction of low-salinity water mass formation. The black solid and dashed lines present the sea surface and mixed layer, respectively. The green arrow denotes the downward Ekman pumping due to negative WSC (${W}_{Ekman}$), and the red arrow denotes the downward velocity at the base of the mixed layer caused by the AE (${W}_{AE}$) related to the inherent topographic effect of the XS (black area). The ${W}_{b}$ presents the vertical velocity of water parcel at the base of the mixed layer. The figure was plotted using MATLAB R2012a (http://www.mathworks.com/).

Subduction of a low-salinity (~34.32) water mass is found in spring (especially in April) near the Xisha Islands (XS) in the SCS based on climatologically observed temperature and salinity datasets, and with a spatial scale of at least 200 km. The relatively homogeneous low-salinity water mass mainly subducts from the surface layer into the intermediate layer, accompanied by a thermocline ventilation, and a relatively low potential vorticity (PV) tongue vertically extending from the bottom of the mixed layer to the thermocline. Dynamical analyses show that the subduction formation is mainly dominated by vertical motion (Fig. 6), including downward Ekman pumping due to negative WSC (${W}_{Ekman}$), and downward velocity caused by an AE related to the inherent topographic effect of the XS (${W}_{AE}$).

The low-salinity water mass is similar to the classic mode water in the open oceans such as the North Pacific (NP), except for lacking of homogeneous temperature and density, which may be related to strong diapycnal mixing process around island chains^33^. The classic mode water is defined by nearly vertically homogenous temperature, salinity, density and low PV water mass within the main thermocline^34,35^. In the northern SCS, there is large heat loss in winter which may favor the thermocline ventilation (Fig. S9). Therefore, to some extent the low-salinity water mass subduction can be appropriately called as a deformed mode water in the SCS (SCSDMW), which is a special kind of mode water. Physical oceanographers have rarely reported mode water in the SCS, which may be related to that it has deformation and hard to be observed when diagnosing by the classic definition of mode water. In the NP, the large-scale subduction of mode water is mainly dominated by the lateral induction and Ekman pumping, while small-scale subduction caused by eddies play a significant role on the total subduction^19^. In the SCS, although the mixed layer is too shallow to produce remarkable lateral induction, downward vertical motion caused by Ekman pumping and an AE provides favorable conditions for the SCSDMW subduction in April.

As the SCS is a tropical basin where the precipitation is in general larger than evaporation, the sea surface salinity is mostly lower than 34.0 south of 18°N and fresher than the XS low-salinity water (>34.3) beneath the thermocline. It is worth noting that there is a northeastward flow away from east of Vietnam in February and April, which may carry coastal low-salinity water to the XS (Fig. S10). Different from April, in February the northeastward flow appears south of the XS, which is unfavorable for the AE formation induced by flow-seamount interactions and thus the low-salinity water mass subduction (Fig. 3b).

In our study, we focus on presenting the features of the low-salinity water mass subduction and preliminarily analyzing the corresponding dynamic mechanism. These results have some implications for the circulation structures and oceanic uptake of anthropogenic carbon in the SCS. Work is underway to further investigate the dynamic processes for the low-salinity water mass subduction based on numerical experiments, including topography, WSC, river runoff, precipitation and vertical mixing coefficient.

## Methods

Estimations of geostrophic currents and relative vorticity. We calculate the geostrophic current structures based on the GDEM-V3 dataset by using the thermal wind relation^13^ which is defined as:

2 $$u=-\frac{g}{f{\rho }_{0}}{\int }_{{z}_{0}}^{z}\frac{\partial \rho }{\partial y}dz$$

3 $$v=\frac{g}{f{\rho }_{0}}{\int }_{{z}_{0}}^{z}\frac{\partial \rho }{\partial x}dz$$

where *g* is the gravitational acceleration, *f* is the Coriolis parameter, *ρ* is the potential density, *ρ*_0_ is the reference density (1024 kg m^−3^) and *z*_0_ is the reference level (2000 m). The general circulation pattern presented in this study is not sensitive to the choice of reference level. In equations (2) and (3), *u* and *v* are the eastward and northward components of the geostrophic currents at depth *z*, respectively.

### Preliminary analyses of subduction rate

According to the work of Willams^15^, the water mass subduction rate (${S}^{wm}$) is defined as the volume flux of fluid per unit area entering the thermocline from the mixed layer:

4 $${S}^{wm}=-(\frac{\partial h}{\partial t}+{U}_{b}\cdot \nabla h+{W}_{b})$$

where (${U}_{b}$, ${W}_{b}$) is the velocity of water parcel at the base of the mixed layer (Z = −h), $\frac{\partial h}{\partial t}$ the rate of the mixed layer shallowing, and ${U}_{b}\cdot \nabla h$ the lateral induction. The SCS has shallower MLD, which results in the contributions of MLD variations to the ${S}^{wm}$ can be neglected to some extent (Fig. 5), that is ${S}^{wm}\approx -{W}_{b}$. For large-scale subduction, the ${W}_{b}$ is calculated by the Ekman pumping rate (${W}_{Ekman}$). But in an eddying ocean, there is an additional subduction caused by eddy^19^, when separating the fluid variables into “mean” and “eddy” components. Therefore, the ${S}^{wm}$ in present study can be simply estimated by:

5 $${S}^{wm}\approx -{W}_{b}\approx -({W}_{Ekman}+{W}_{AE})$$

where ${W}_{Ekman}$ is the Ekman pumping rate caused by WSC, and ${W}_{\mathrm{AE}}$ is the vertical velocity at the base of the mixed layer related to the AE.

### Data Availability

#### Temperature and salinity data

We collected three datasets of monthly climatologically observed temperature and salinity. They are the U. S. Navy Generalized Digital Environment Model version 3 (GDEM-V3, http://www.usgodae.org/pub/outgoing/static/ocn/gdem/)^20^, the SCS Physical Oceanographic dataset (SCSPOD14, https://www.nature.com/sdata/)^21^ and the World Ocean Atlas 2013 (WOA13, https://www.nodc.noaa.gov/OC5/woa13/)^22,23^, respectively. All of these datasets have a horizontal grid resolution of 0.25° × 0.25°. The GDEM-V3 was edited by the Naval Research Laboratory, with a vertical grid of 78 standard layers ranging from the surface to 6600 m. The detail description of GDEM-V3 can be referenced to Carnes^20^. The WOA13 was a set of objectively analyzed fields at standard depth levels produced from the Word Ocean Database (WOD) and the Real-time Geostrophic Oceanography (Argo) project^22,23^. Compared with the WOA13, the SCSPOD14 was added by about 10000 profiles performed by 203 cruises from the South China Sea Institute of Oceanology (SCSIO) between 1971 and 2014^21^, and the GDEM-V3 was added lots of measurements observed by the U.S. Navy, in terms of temperature and salinity profile numbers. Different from the WOA13 and SCSOD14, the GDEM-V3 eliminated interpolation across land boundary.

#### Satellite altimetry and wind field products

The merged satellite altimeter products during the period of 1993–2016, with a horizontal grid resolution of 0.25° × 0.25°, obtained from the Archiving, Validation, and Interpretation of Satellite Oceanographic data (AVISO; http://www.aviso.oceanobs.com), are used to assess the surface geostrophic currents. The Quick SCATterometer products during 2000–2008 (http://www.remss.com/missions/), with a grid space of 0.25°, are applied to calculate the WSC and Ekman pumping rate.

#### Heat flux datasets

The net shortwave radiation (SW) and longwave radiation (LW) are from the International Satellite Cloud Climatology Project (ISCCP; https://isccp.giss.nasa.gov/outgoing/FLUX/), the latent heat flux (LH) and sensible heat flux (SH) are distributed by the Objectively Analyzed air-sea Fluxes (OAFlux; http://oaflux.whoi.edu). The monthly SW, LW, LH and SH with 1.0° × 1.0° resolution are collected during 1984–2006. Net heat flux (Q_net_) is computed by Q_net_ = SW-LW-LH-SH.

## Electronic supplementary material

Supplementary Information
