# Supplementary material for: Subduction of a low-salinity water mass around the Xisha Islands in the South China Sea
Source: Sci Rep. 2018 Feb 15;8:3074. doi: 10.1038/s41598-018-21364-3 (PMC5814407; doi:10.1038/s41598-018-21364-3)
Supplement: Supplementary file 1 — Supplementary Information [file 41598_2018_21364_MOESM1_ESM.pdf]

Supplementary Information:

Subduction of a low-salinity water mass around the Xisha Islands in the South China Sea

Zhida Huang[1,2], Wei Zhuang[1], Hailong Liu[3,4], & Jianyu Hu[1]

[1]State Key Laboratory of Marine Environmental Science, College of Ocean and Earth Sciences, Xiamen University, Xiamen 361102, China.

[2]Key Laboratory of Coastal and Wetland Ecosystems of Ministry of Education, College of the Environment and Ecology, Xiamen University, Xiamen 361102, China.

[3]State Key Laboratory of Numerical Modeling for Atmospheric Sciences and Geophysical Fluid Dynamics, Institute of Atmospheric Physics, Chinese Academy of Sciences, Beijing 100029, China.

[4]College of Earth Sciences, University of Chinese Academy of Sciences, Beijing 100049, China.

Corresponding author address: Dr. J. Hu and Dr. W. Zhuang, College of Ocean and Earth Sciences, Xiamen University, Xiamen 361102, China.

Email: hujy@xmu.edu.cn; wzhuang@xmu.edu.cn

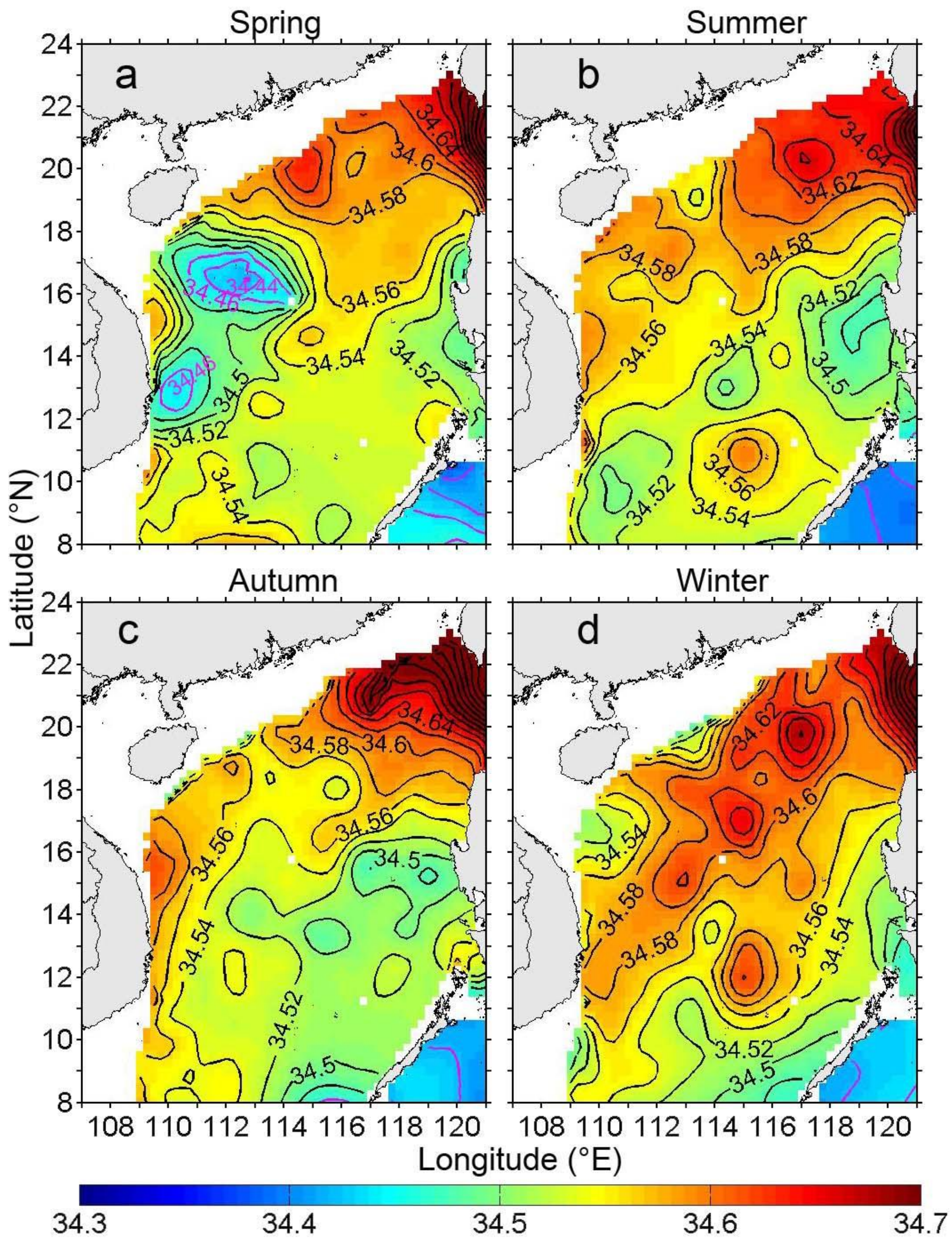

Figure S1. Same as Fig. 2, except for the subsurface layer (150 m). The figure was made using MATLAB R2012a (http://www.mathworks.com/).

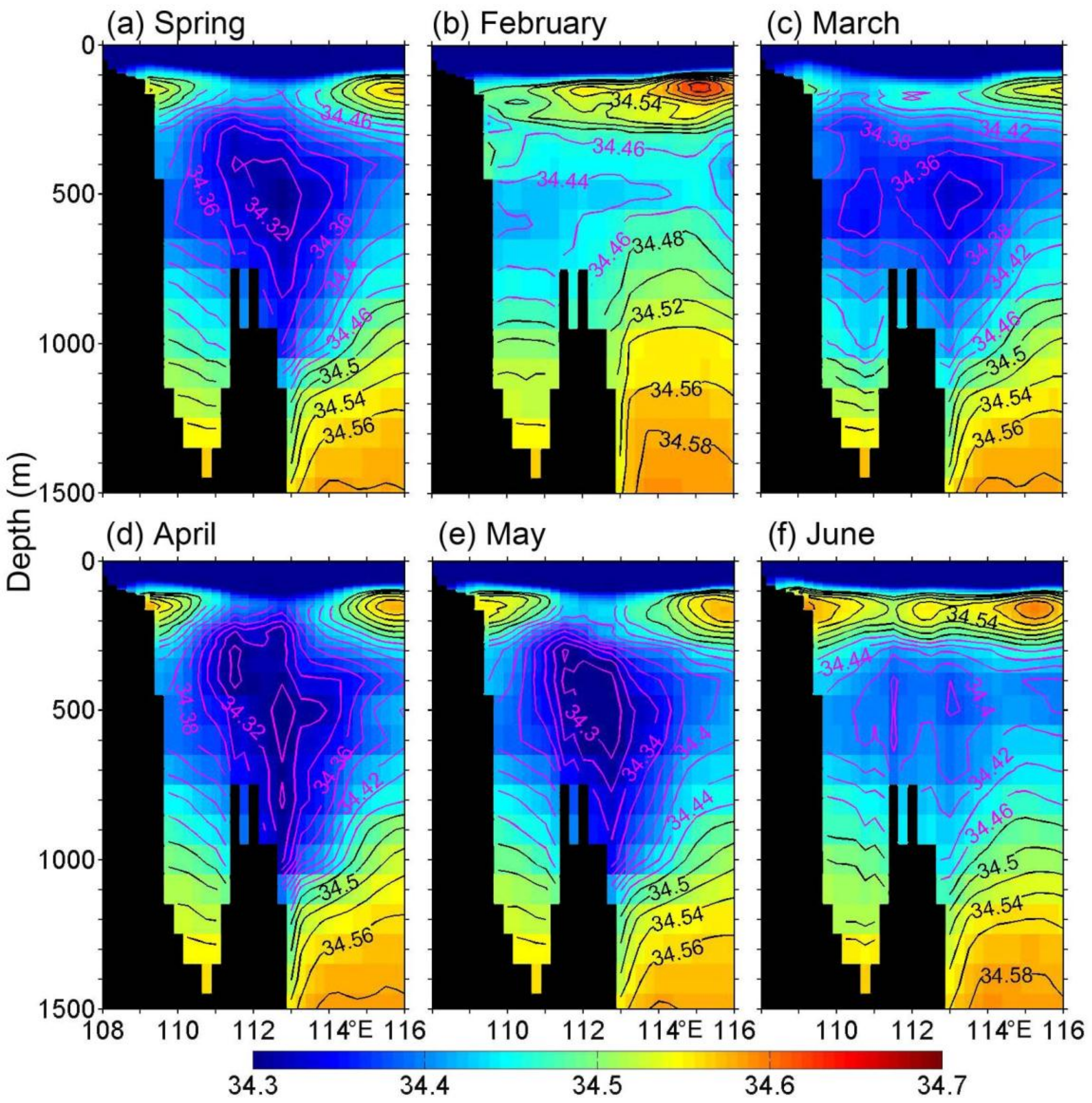

Figure S2. As in Fig. 3, except for sectional distributions of salinity for Transect B. The black shades indicate the topography extracted from the GDEM-V3 dataset[20]. The figure was made using MATLAB R2012a (http://www.mathworks.com/).

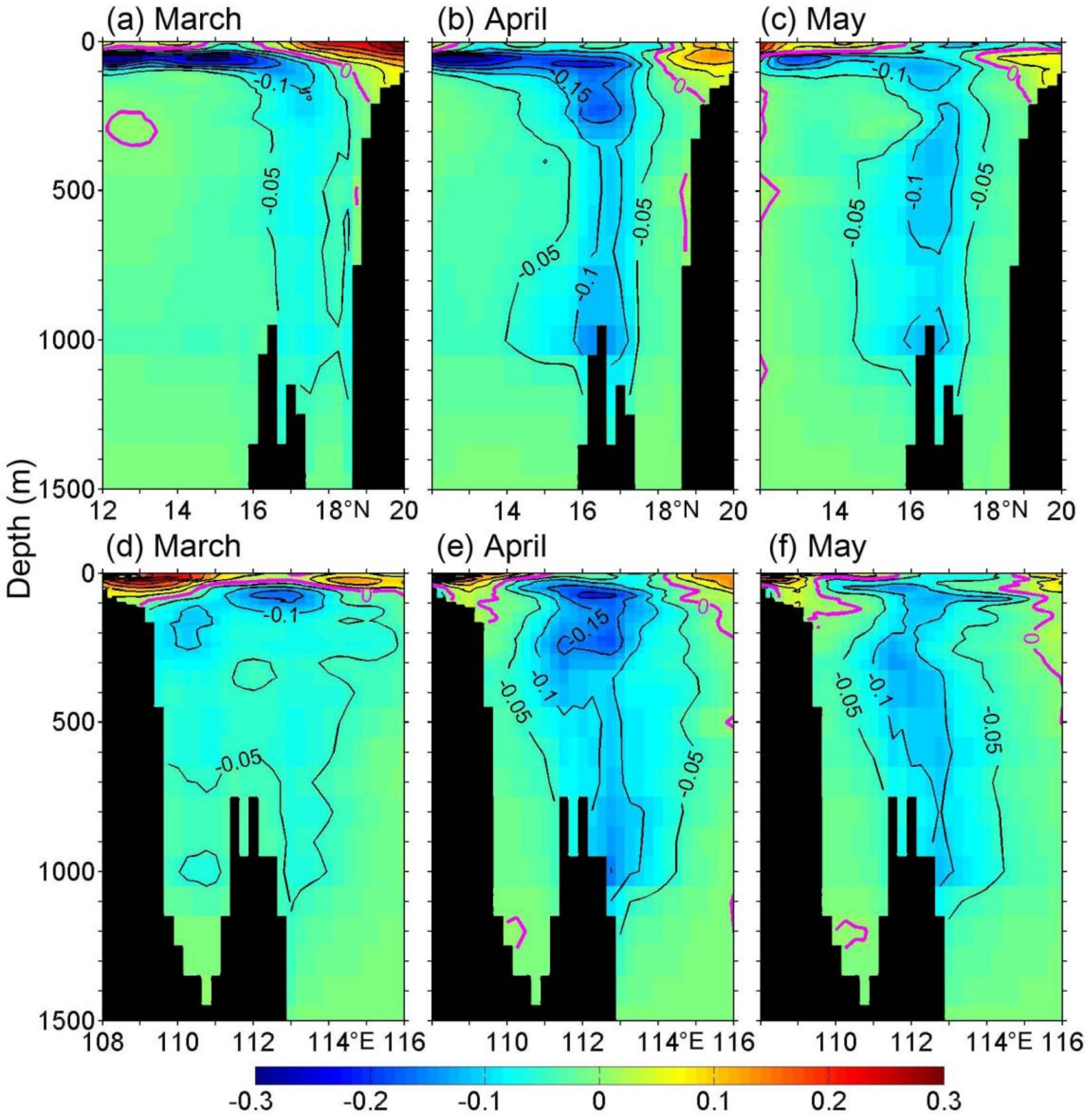

Figure S3. Monthly salinity anomalies for Transect A in (**a**) March, (**b**) April and (**c**) May, respectively. Same as (**a–c**), (**d–f**) except for Transect B. The monthly anomaly is subtracted from the annual average. The pink curves highlight the 0 isohaline. The black shades indicate the topography extracted from the GDEM-V3 dataset[20]. The figure was generated using MATLAB R2012a (http://www.mathworks.com/).

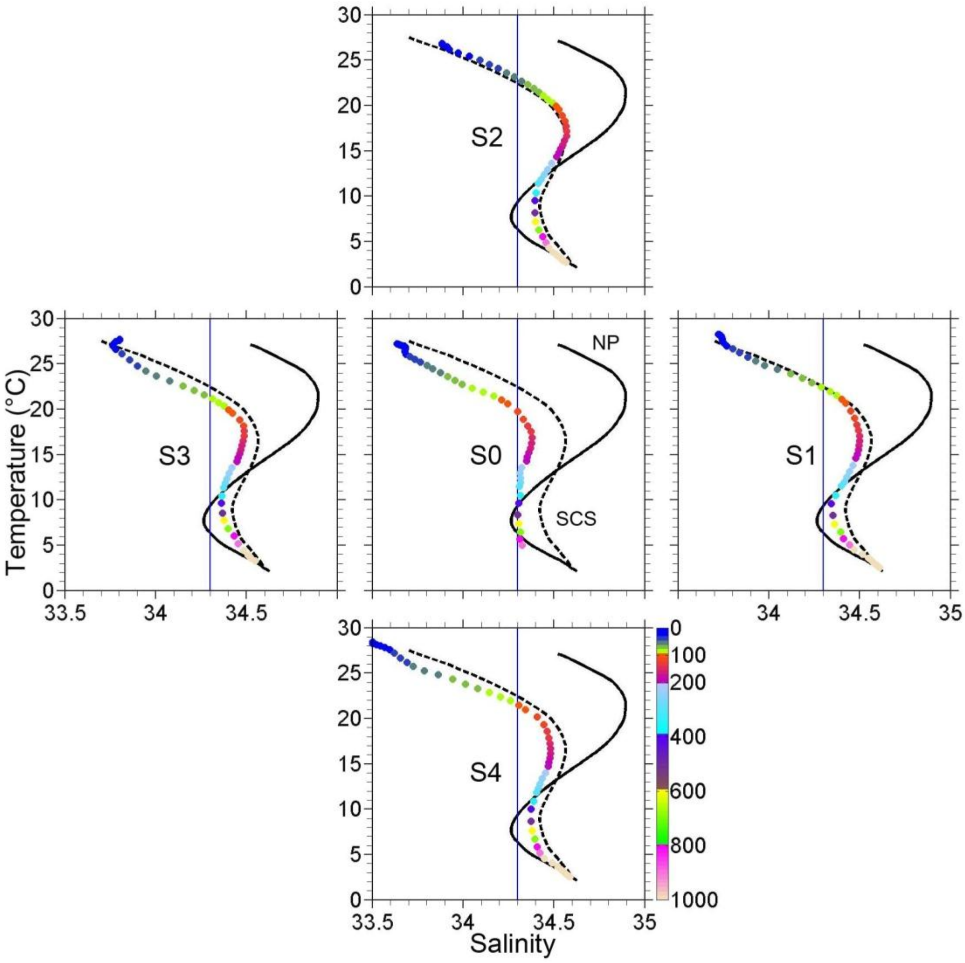

Figure S4. T-S diagrams for five stations S0–S4 (black dots in Fig. 1) in April, filled by depth (m). Station S0 is located at the low-salinity core near the XS (Figs 2a and S1a), and stations S1–S4 have a distance of 2.0 ° from S0 to the east, north, west and south (black dots in Fig. 1), respectively. The black dashed and solid lines are the T-S diagrams for the annual mean SCS water (Box1 in Fig. 1) and the NP water (Box2 in Fig. 1) based on the GDEM-V3 dataset[20], respectively. The figure was plotted using MATLAB R2012a (http://www.mathworks.com/).

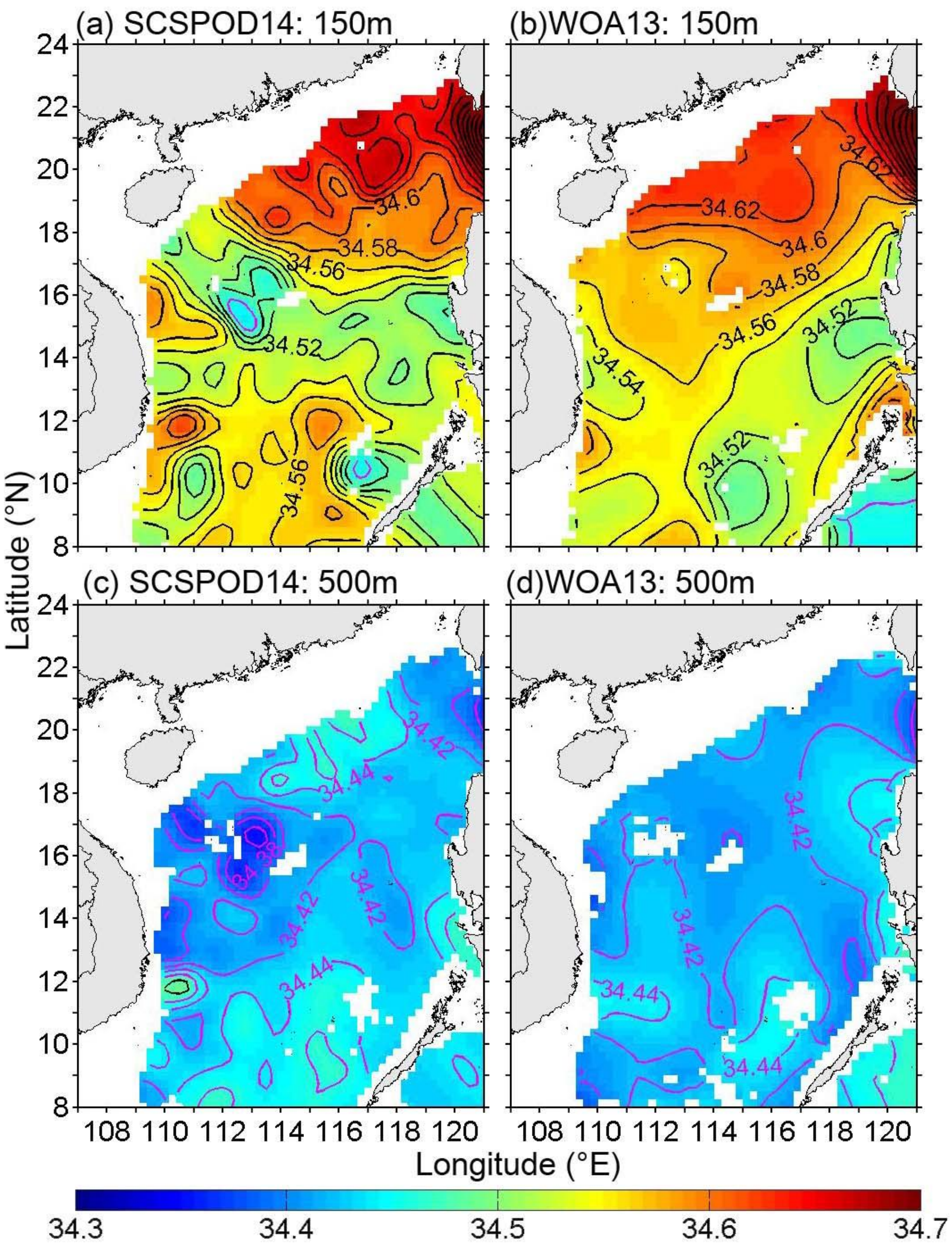

Figure S5. Salinity distributions in spring from the SCSPOD14 and WOA13 datasets. Left panels: subsurface (150 m) and intermediate (500 m) layers based on the SCSPOD14. Right panels: similar as left, except for the WOA13. The figure was made using MATLAB R2012a (http://www.mathworks.com/).

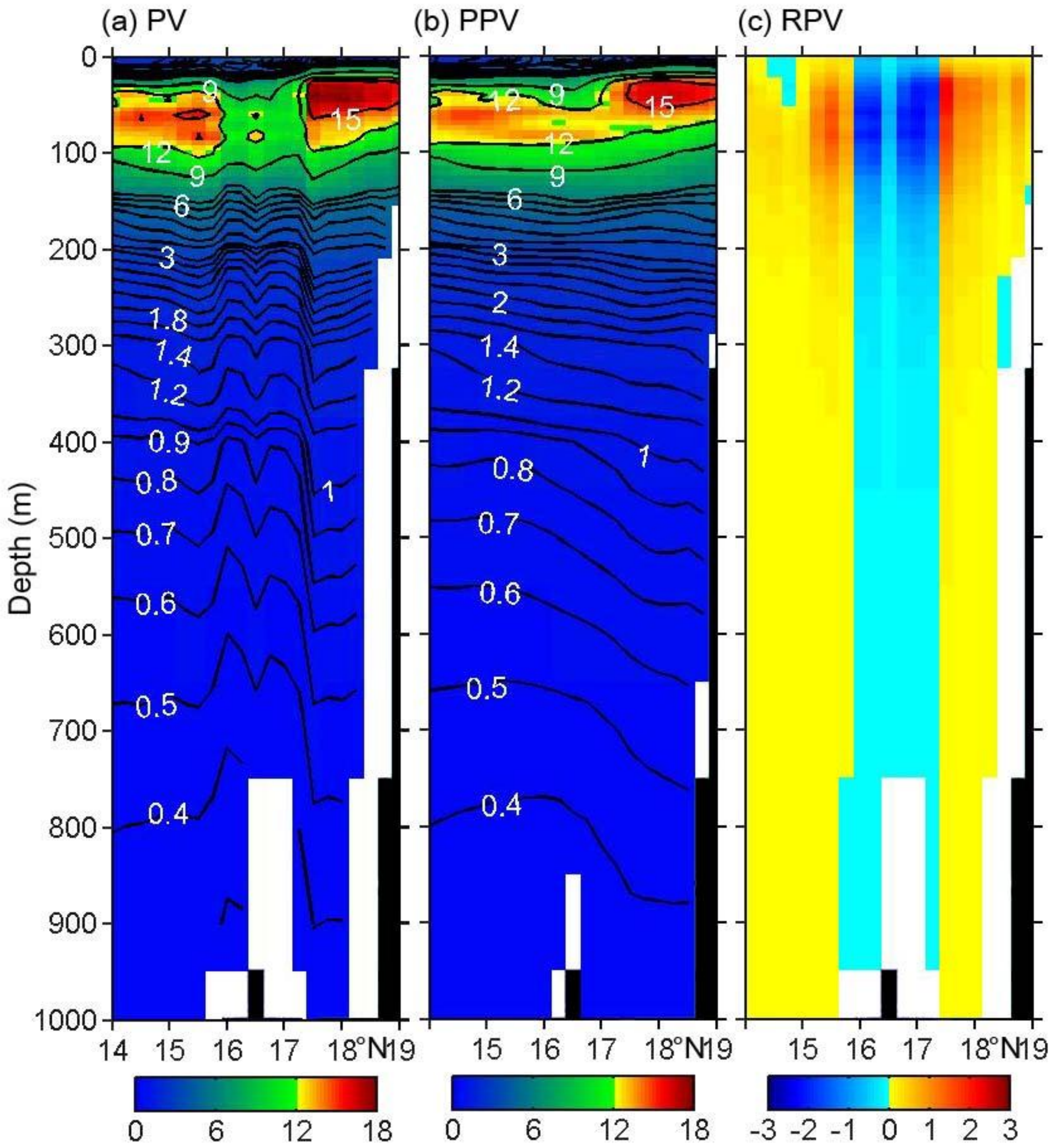

Figure S6. Distributions of (**a**) potential vorticity (PV; $\times10^{-10}$ m^{-1} s^{-1}), (**b**) planetary potential vorticity (PPV) and (**c**) relative potential vorticity (RPV) along Transect C between 0 and 1000 m in April. PV is the sum of PPV and RPV. The black shades indicate the topography extracted from the GDEM-V3 dataset[20]. The figure was made using MATLAB R2012a (http://www.mathworks.com/).

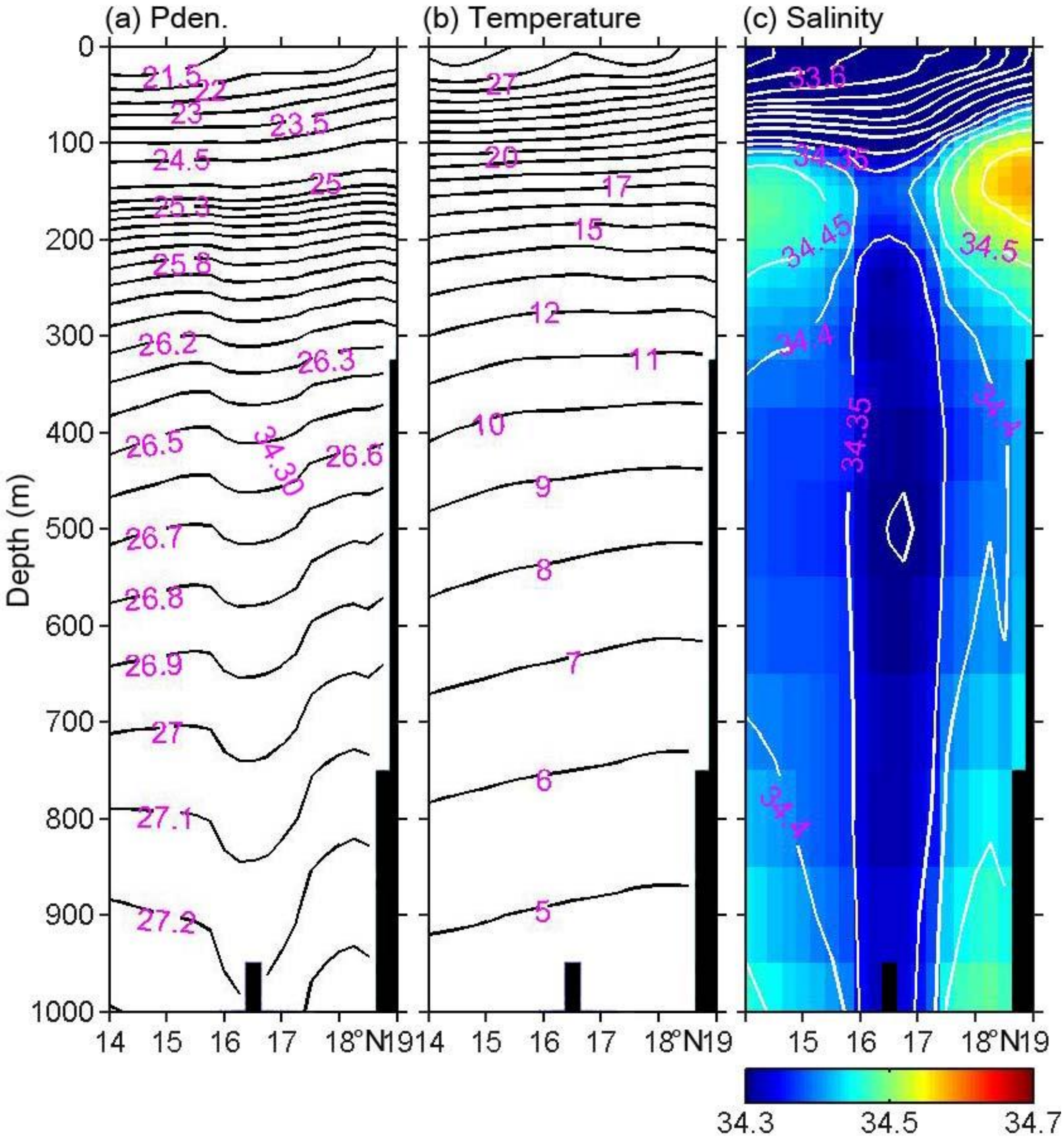

Figure S7. As in Fig. S6, except for (**a**) potential density (kg m^{-3}), (**b**) temperature (℃) and (**c**) salinity. The figure was made using MATLAB R2012a (http://www.mathworks.com/).

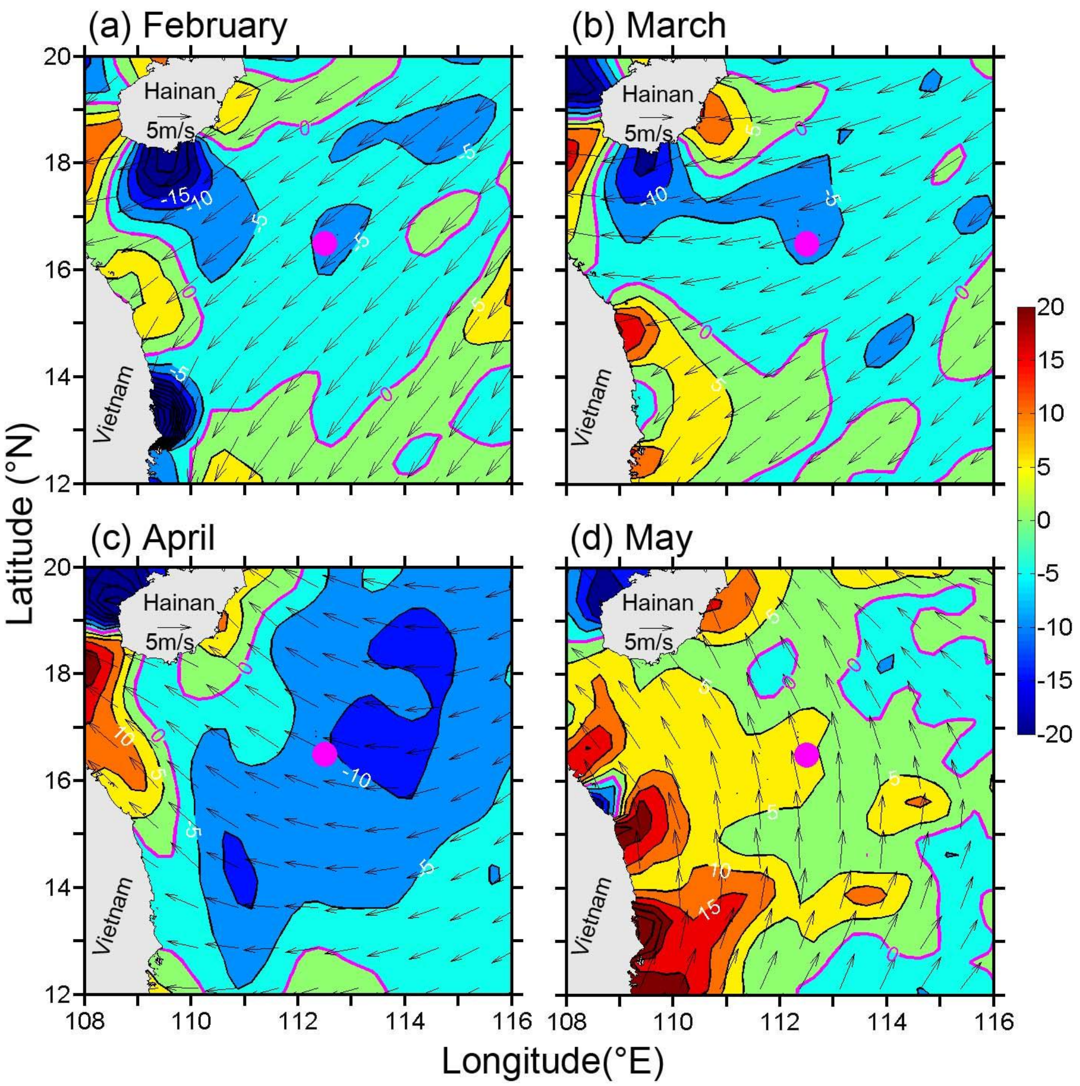

Figure S8. Distributions of the wind stress curl (WSC; shaded in color; $\times 10^{-8}$ N m^{-3}) and wind vectors in (**a**) February, (**b**) March, (**c**) April and (**d**) May, respectively, based on the Quick SCATterometer dataset. The pink curves highlight the contour value of 0. The pink dot denotes the XS. The figure was plotted using MATLAB R2012a (http://www.mathworks.com/).

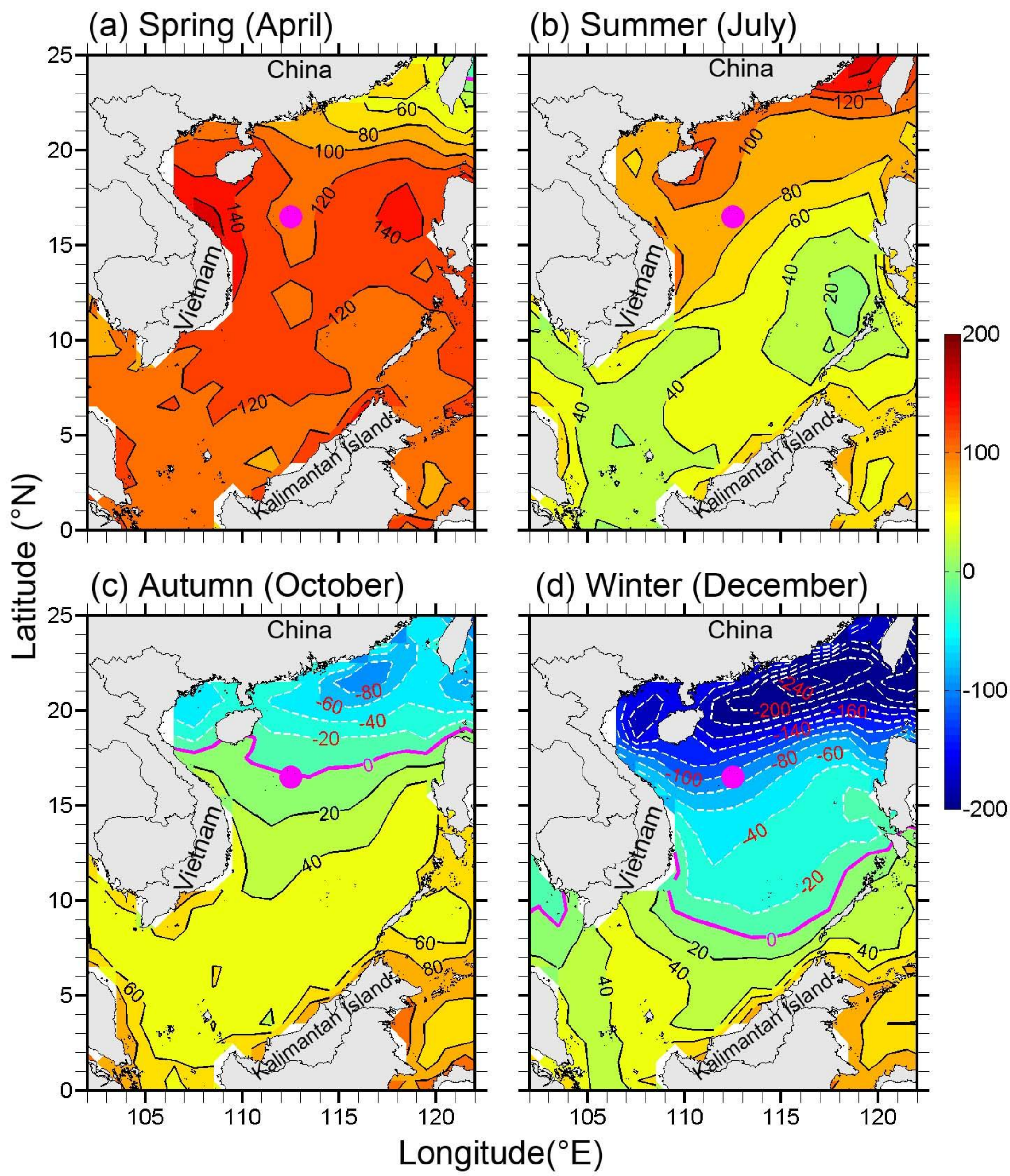

Figure S9. Seasonal distributions of the net downward heat flux (Q_{net}; shaded in color; W m^{-2}) in (**a**) spring (April), (**b**) summer (July), (**c**) autumn (October) and (**d**) winter (December), respectively, based on the combination of OAFlux and ISCCP datasets during 1984–2006. The black solid (white dashed) contours denote the Q_{net} with value greater (less) than 0, the pink curves highlight the contour value of 0. The pink dot denotes the XS. The figure was plotted using MATLAB R2012a (http://www.mathworks.com/).

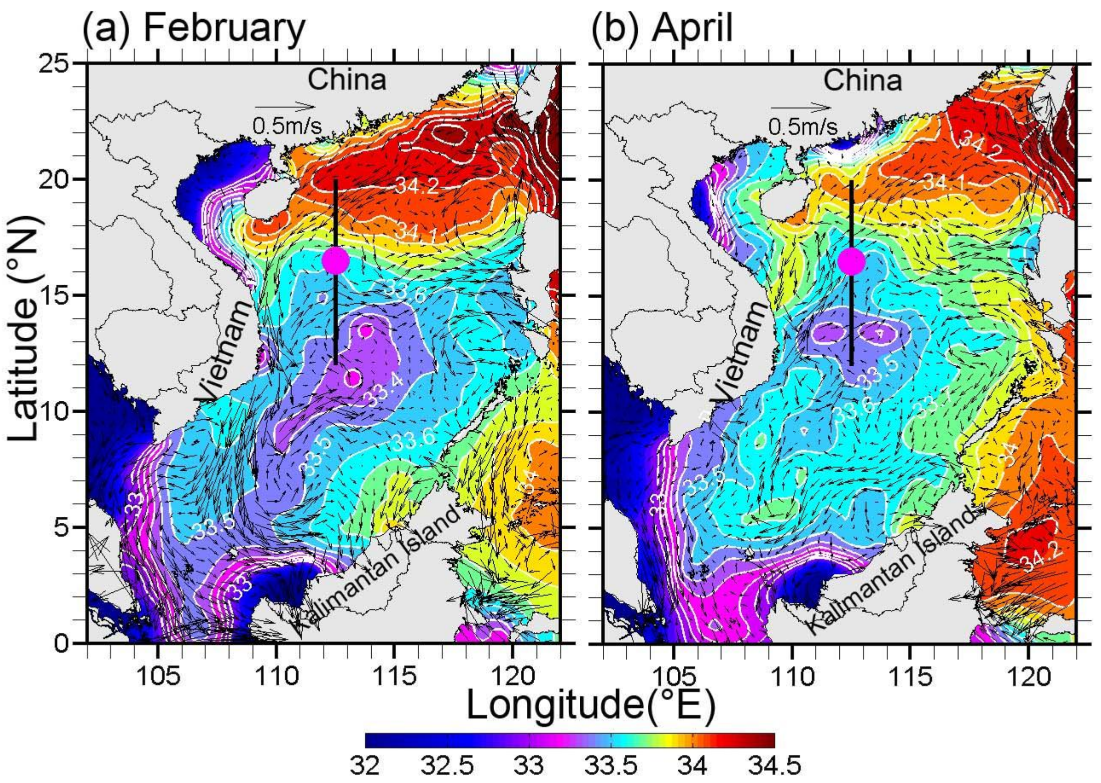

Figure S10. Distributions of the sea surface salinity (SSS; shaded in color) from the GDEM-V3 dataset[20] and geostrophic currents based on AVISO in (**a**) February and (**b**) April, respectively. The black line indicates Transect A shown in Fig.1. The pink dot denotes the XS. The figure was plotted using MATLAB R2012a (http://www.mathworks.com/).
